# Virginia

**Published:** 1896-05

**Authors:** 


*Virginia.* Governor O'Ferral recently appointed the following members on the State Board of Veterinary Examiners: W. H. Harbaugh, V.S., of Richmond; E. P. Niles, D.V.M., of Blacksburg; George C. Faville, D.V.M., of Norfolk; Charles McCullough, D.V.S., of Howardsville; A. J. Burkholder, D.V.S., of Staunton. The appointments, which are for a term of four years each, beginning May 1, 1896, are made under the law passed during the last session of the General Assembly and approved February 27, 1896.

